# Functional effect of mir-27b on myostatin expression: a relationship in piedmontese cattle with double-muscled phenotype

**DOI:** 10.1186/1471-2164-14-194

**Published:** 2013-03-19

**Authors:** Silvia Miretti, Eugenio Martignani, Paolo Accornero, Mario Baratta

**Affiliations:** 1 Department of Veterinary Science, University of Torino, via Leonardo da Vinci 44, Grugliasco, 10095, Italy

**Keywords:** MicroRNA, Bovine, Skeletal muscle, Hypertrophy

## Abstract

**Background:**

In Piedmontese cattle the double-muscled phenotype is an inherited condition associated to a point mutation in the myostatin (MSTN) gene. The Piedmontese MSTN missense mutation G938A is translated to C313Y myostatin protein. This mutation alters MSTN function as a negative regulator of muscle growth, thereby inducing muscle hypertrophy. MiRNAs could play a role in skeletal muscle hypertrophy modulation by down-regulating gene expression.

**Results:**

After identifying a 3^′^-UTR consensus sequence of several negative and positive modulator genes involved in the skeletal muscle hypertrophy pathway, such as IGF1, IGF1R, PPP3CA, NFATc1, MEF2C, GSK3b, TEAD1 and MSTN, we screened miRNAs matching to it. This analysis led to the identification of miR-27b, miR-132, miR-186 and miR-199b-5p as possible candidates. We collected samples of longissimus thoracis from twenty Piedmontese and twenty Friesian male bovines. In Piedmontese group miR-27b was up-regulated 7.4-fold (p < 0.05). Further, we report that the level of MSTN mRNA was about 5-fold lower in Piedmontese cattle vs Friesian cattle (p < 0.0001) and that less mature MSTN protein was detected in the Piedmontese one (p < 0.0001). Cotransfection of miR-27b and psi-check2 vector with the luciferase reporter gene linked to the bovine wild-type 3^′^-UTR of MSTN strongly inhibited the luciferase activity (79%, p < 0.0001).

**Conclusions:**

These data demonstrate that bovine MSTN is a specific target of miR-27b and that miRNAs contribute to explain additive phenotypic hypertrophy in Piedmontese cattle selected for the MSTN gene mutation, possibly outlining a more precise genetic signature able to elucidate differences in muscle conformation.

## Background

Skeletal muscle hypertrophy is defined as an increase in muscle mass, which in the adult animal comes as a result of an increase in size of skeletal muscle fibers. In the last years different mechanism of action have been reported to regulate muscle hypertrophy. The major extracellular mediator of skeletal muscle hypertrophy is thought to be Insulin Growth Factor-1 (IGF-1) which binds to its receptor IGF1R to initiate a cascade of signaling pathways via phosphoinositide 3-kinase (PI3-K/Akt/mammalian target of rapamycin (mTOR)) [1,2]. However, several lines of evidence suggest that IGF-1 also mediates hypertrophy through calcineurin (PPP3CA)/nuclear factor of activated T-cells (NFAT) signaling pathway [3]. Moreover, studies suggested that myocyte enhancer factor 2C (MEF2C) regulates the hypertrophic process [4]. Also glycogen synthase kinase 3 beta (GSK3b), that is a distinct substrate of Akt, has been shown to modulate hypertrophy. Through Akt phosphorylation, GSK3b activity is inhibited [5] and its inhibition may induce hypertrophy by stimulating protein synthesis independent of the mTOR pathway. TEAD1 (transcription enhancer factor 1) regulates the expression of many skeletal muscle-specific genes [6]. TEAD1 is a member of the TEA domain family and is constitutively expressed in cardiac and skeletal muscles in pigs, mice and humans [7]. The transcriptional regulation of TEAD1 to muscle-specific genes is implemented by co-operation with numerous co-factors, including MEF2 [8]. Myostatin (MSTN) is a member of the transforming growth factor-b (TGF-b) superfamily of secreted growth and differentiation factors [9]. In Piedmontese cattle the double-muscled phenotype is an inherited condition associated to a point mutation in the MSTN gene. The Piedmontese MSTN missense mutation G938A is translated to C313Y myostatin protein with the consequent loss of one of the disulphide bonds (C313-C374) involved in the characteristic TGF-b family cystine-knot structural motif [10]. This mutation alters the function of MSTN as a negative regulator of muscle growth, thereby inducing muscle hyperplasia and hypertrophy. This breed has been systematically selected for double muscling to the point of fixation in many herds (>96% homozygosis in the Piedmonte Region in Italy), but some difference in muscularity phenotype is still present. MicroRNAs (miRNAs) are small non-coding RNA molecules (20–25 nucleotides), highly conserved, that regulate gene expression through binding with imperfect complementarities sequences of messenger RNA (mRNA) [11]. It is becoming increasingly evident that miRNAs regulation of mRNAs represents an effective way of interfering with function by acting at protein translation level. This takes place when the miRNA targets the 3^′^- untranslated region (3^′^-UTR) of transcripts by imperfect base pairing and functions to inhibit translation. In some cases, degradation of the target mRNA is also a mechanism or a consequence of miRNA-mediated suppression [12]. MiRNAs have been shown to play critical roles in skeletal muscle development as well as in regulation of muscle cell proliferation and differentiation [13]. By down-regulating gene expression, miRNAs could play a role in skeletal muscle hypertrophy modulation. In this study, we have analyzed miRNAs engaged in post-transcriptional regulation of negative or positive modulators involved in the muscular hypertrophy pathway in Piedmontese cattle. In particular, we aimed to investigate on the specific miRNAs involved in MSTN regulation. We screened the 3^′^-UTR matching miRNAs of several genes, such as IGF1, IGF1R, PPP3CA, NFATc1, MEF2C, GSK3B, TEAD1 and MSTN using a computational approach. This analysis led to the identification of miR-27b, miR-132, miR-186 and miR-199b-5p as possible candidates implicated in bovine skeletal muscle hypertrophy. Finally, we report the functional evidence that miR-27b is able to down-regulate MSTN gene in vitro.

## Results

### Expression of IGF1, IGF1R, PPP3CA, NFATc1, MEF2C, GSK3b, TEAD1 and MSTN mRNA in bovine skeletal muscle specimens

**Figure 1:**
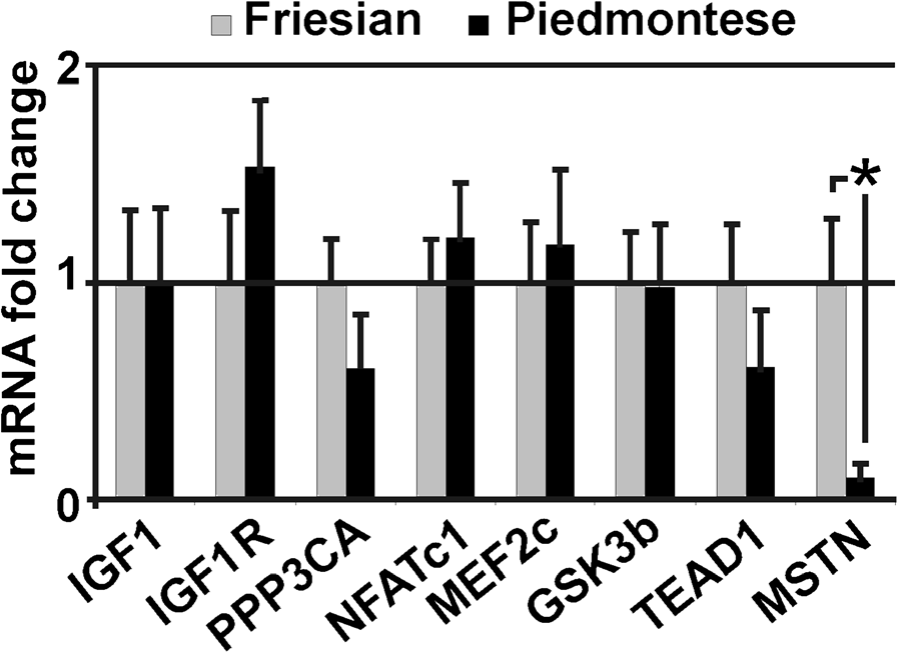
**qPCR of genes correlated with hypertrophy.** The expression mRNA screening of several genes linked with hypertrophy pathway highlights MSTN downregulation of 4.9-fold in Piedmontese skeletal muscle samples. Expression were normalized to the endogenous control, HPRT, and data are shown as fold change mean ± S.D. (n = 20) for each gene compared to Friesian samples. * means values with significant difference (at least p < 0.05).

Skeletal muscle samples were collected from cattle from two breeds with opposite carcass conformation: Piedmontese and Friesian bovines classified respectively as “E” and “O” muscle profile (see Methods). In order to identify possible gene expression differences linked to skeletal muscle phenotype between the two breeds, we have analyzed IGF1, IGF1R, PPP3CA, NFATc1, MEF2C, GSK3b, TEAD1 and MSTN genes using real-time quantitative PCR. As shown in Figure 1, only MSTN mRNA expression displayed a strong difference between the two breeds. In particular, in Piedmontese samples, MSTN mRNA expression was downregulated 4.9-fold compared with Friesian muscle (p < 0.05). On the other hand, the expression of IGF1, IGF1R, PPP3CA, NFATc1, MEF2C, GSK3b and TEAD1 were not significantly different.

### MSTN protein quantification

**Figure 2:**
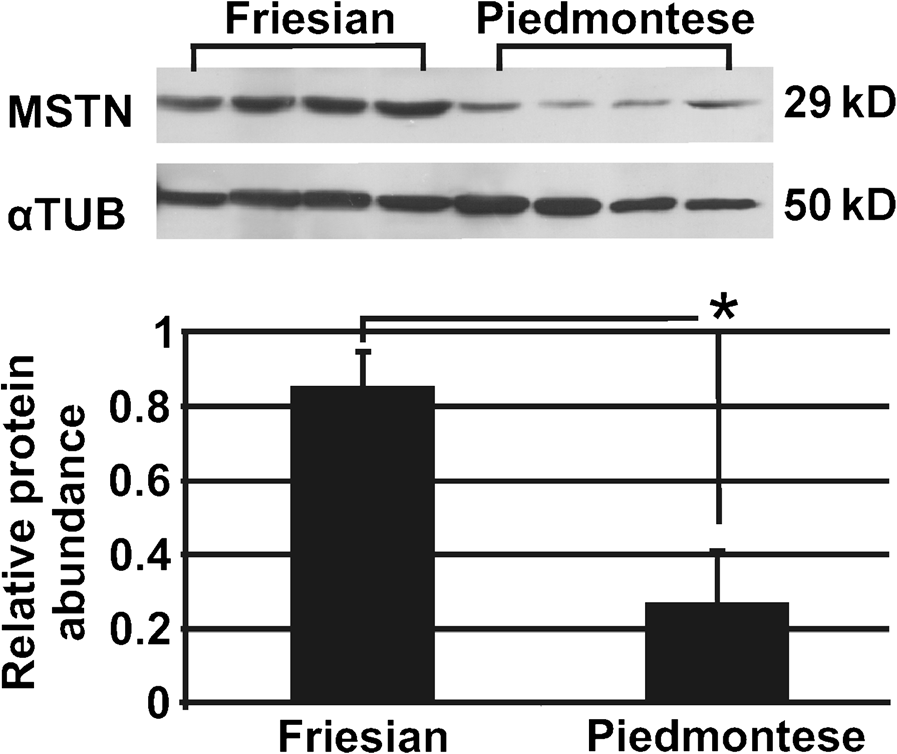
**MSTN protein abundance analysis.** Representative Western blot analysis of mature MSTN from longissimus thoracis in Friesian and Piedmontese bovines. Alpha-tubulin is shown as a loading control. Bar graphs shows densitometry quantification of blots from MSTN normalized to alpha-tubulin. Relative expression differences are shown as densitometry mean ± S.D. (n = 20) * means values with significant difference (at least p < 0.05).

Total proteins were extracted from the muscle samples and measured by Western blot analysis. As shown in Figure 2, MSTN specific antibody recognized a ~29-kDa mature MSTN in both Friesian and Piedmontese cattle muscle extracts. Alpha-tubulin was used as loading control. Densitometric analysis of Western blot indicates that in Piedmontese breed there was less abundance MSTN than in Friesian one (p < 0.0001) (Figure 2).

### Targeting site of miRNAs in IGF1, IGF1R, PPP3CA, NFATc1, MEF2C, GSK3B, TEAD1 and MSTN 3^′^-UTR

To identify miRNAs which could modulate bovine skeletal muscle hypertrophy, we have screened miRNAs which potentially suppress expression of genes involved as negative or positive modulators in the muscular hypertrophy pathway. For this purpose, miRNAs target site prediction for bovine IGF1, IGF1R, PPP3CA, NFATc1, MEF2C, GSK3b, TEAD1 and MSTN were performed with the use of TargetScan 5.1 algorithms (http://www.targetscan.org). This analysis combined with bibliographic data research induced us to investigate miR-27b, miR-132, miR-186 and miR-199b-5p as potential candidates implicated in bovine skeletal muscle hypertrophy. The number of gene targets resulted higher for miR-27b than for other miRNAs (Table 1).

**Table 1:** miRNAs and putative target genes

**MiRNA**	**Conserved targets**	**Gene name**	**Number of 3**^′^**-UTR conserved site**	**Gene rank on basis of total context score**
miR-27b	1106	IGF1	1	1089
		NFATc1	1	224
		MEF2C	1	885
		GSK3b	1	905
		TEAD1	3	420
		MSTN	1	82
miR-132	389	PPP3CA	1	383
		GSK3b	1	374
miR-186	777	IGF1	1	508
		IGF1R	2	349
miR-199b-5p	460	GSK3b	2	103

TargetScan predicts biological targets of miRNAs by searching for the presence of conserved 8mer and 7mer sites that match the seed region of each miRNA. In mammals, predictions are ranked based on the predicted efficacy of targeting as calculated using the context scores of the sites. The context score is the sum of the contribution of these four features: site-type contribution; 3^′^ pairing contribution; local AU contribution; position contribution.

### Expression of miR-27b, miR-132, miR-186 and miR-199b-5p in bovine skeletal muscle

**Figure 3:**
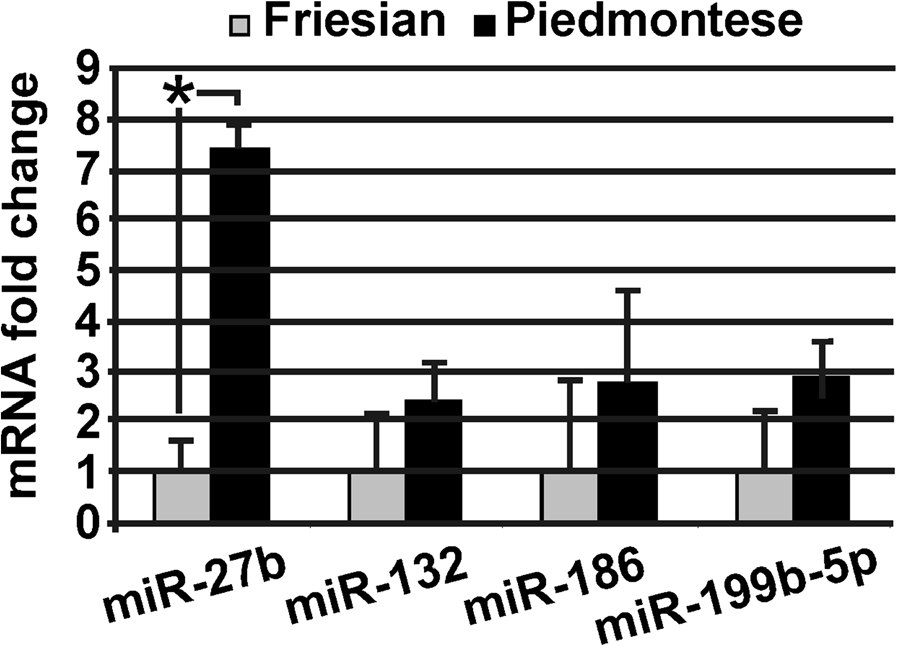
**Expression of miRNAs targeting IGF1, IGF1R, PPP3CA, NFATc1, MEF2C, GSK3b, TEAD1 and MSTN.** MiR-27b is upregulated 7.4-fold in Piedmontese breed. miR-27b, miR-132, miR-186 and miR-199b-5p were screened in TaqMan real-time PCR and expression was normalized to the endogenous control, miR-16. Relative expression differences are shown as fold change mean ± S.D. (n = 20) for each miRNAs compared to Friesian samples. * means values with significant difference (at least p < 0.05).

To examine which, if any, of the miRNAs identified from the TargetScan algorithm were expressed in bovine muscle, we used microRNA TaqMan Assay. Sample of both breeds mir-27b, miR-132, miR-186 and miR-199b-5p were detectable. No significant difference were observed for miR-132, miR-186 and miR199b-5p expression. On the contrary, miR-27b was shown significantly upregulated with a 7.4-fold increase in Piedmontese cattle (p < 0.0001) (Figure 3).

As a consequence of the data shown above, in Piedmontese breed samples, we observed an inverse relationship between MSTN transcript and miR-27b expression. This observation was confirmed by Spearman correlation test (ρ = −0.70; 95% confidence interval: -0.87, -0.37; p < 0.001). A correlation was also carried out between MSTN abundance protein and miR-27b expression. Also in this case results showed a significant correlation (ρ = −0.59; 95% confident interval: -0.85, -0.12; p < 0.02).

The data of MSTN expression downregulation in mRNA and protein analysis, and the upregulation of miR-27b in Piedmontese bovine samples prompted us to verify if MSTN was a direct target of miR-27b in a functional assay.

### The 3^′^-UTR of bovine myostatin mRNA is a direct target of miR-27b

**Figure 4:**
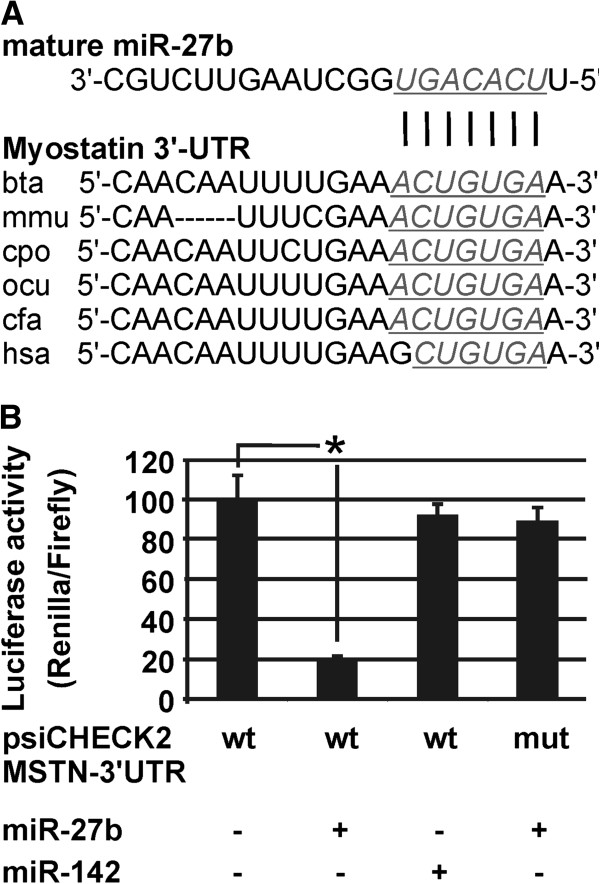
**miR-27b directly targets MSTN.** (**A**) Sequence alignment of putative miR-27b-binding site in the MSTN 3^′^-UTR, showing a high level of complementarity and sequence conservation among mammalian species. The wild type bovine MSTN mRNA sequence is shown with potential binding sites underlined. (**B**) Luciferase assay was performed to test whether MSTN is a bona fide target of miR-27b. 293 T cells were co-transfected with psi-check2 reporter vectors containing native or mutated MSTN 3^′^-UTR, and miR-27b mimics or miRNA mimics negative control (miR-142), followed by cell lysis and luciferase assays 48 h later. Mutation of the miR-27b recognition sequence abolished responsiveness to miR-27b co-transfection compared with the wild-type construct. Psi-check2 was used as negative controls. * means values with significant difference (at least p < 0.001).

Through a target prediction algorithm (TargetScan), we identified MSTN as the potential target of miR-27b. TargetScan predicts 1106 conserved targets for miR-27a/b in the cow, of which MSTN is ranked 82th on the basis of total context score. To determine whether MSTN is a direct target of miR-27b, we performed luciferase reporter assays in 293 T cells. Co-transfection of pre-miR-27b with the luciferase reporter gene linked to the wild-type 3^′^-UTR of MSTN strongly inhibited the luciferase activity (79%, p < 0.0001) (Figure 4B). Moreover, no effect was observed with the mutant construct of MSTN 3^′^-UTR in which miR-27b seed sequence was mutated. Co-transfection of pre-miR-142 (control miRNA; not complementary to the 3^′^-UTR of MSTN) with the wild-type MSTN 3^′^-UTR construct did not alter the luciferase activity, indicating that miR-27b specifically targeted MSTN (Figure 4B).

## Discussion

Considerable progress has been made in elucidating signalling mechanisms of skeletal muscle hypertrophy at the molecular level, but fine regulatory processes are still unknown. Piedmontese breed has been systematically selected for double-muscled phenotype and the peculiar muscular hypertrophy have been analyzed for a long time. Until now, this hypertrophy was traced back to MSTN point mutation [14]. Here we demonstrate that MSTN is further regulated at post-transcriptional level by miR-27b. In fact, despite the MSTN homozygosis mutation is present in more than 96% of Piedmontese cattle in Piedmonte area, in the North-western part of Italy, some differences in muscle phenotype conformation still exist. It has been previously observed that these differences are visible in animals submitted to homogeneous management conditions (feeding, housing, health status). Therefore, observed hypertrophy variations among animals are related to genetics and they are hereditable, and not linked to the environment (ANABORAPI report) [15]. To understand witch other mechanisms, other than MSTN mutation, could be involved in the Piedmontese muscle phenotype we have analyzed several negative and positive regulator genes involved in the skeletal muscle hypertrophy pathway (IGF1, IGF1R, PPP3CA, NFATc1, MEF2C, GSK3b, TEAD1, MSTN) and relative miRNAs with conserved site on their 3^′^UTR in this breed and in Friesian breed used as control, for opposed carcass conformation. According to our analysis, MSTN, in addition to mutation showed a strong downregulation at mRNA and protein levels. We also observed a significant, high upregulation for miR-27b. Recently, much attention has been focused on the potential role of miRNAs in gene expression regulation. miRNAs may contribute to phenotypic variability in cattle selected for the MSTN gene mutation. In Texel sheep, the MSTN allele is characterized by a G vs A transition in the 3^′^UTR that creates a target site for miR-1 and miR-206, which are highly expressed in skeletal muscle. This causes inhibition of the MSTN gene and contributes to muscular hypertrophy [16]. Analysis of human and murine single nucleotide polymorphism demonstrates that mutations creating or destroying putative miRNA target sites are abundant and therefore might be important sources of phenotypic variation [17]. Current data on the role of miRNAs in myogenesis has been obtained largely from studies on muscle-specific miR-1, miR-133 and miR-206. We have previously demonstrated that Piedmontese and Friesian female, but not male cattle showed differential expression of miR-206 in skeletal muscle leading to hypothesize a relationship between miRNA expression and sex [18]. Here, we screened in males that exhibit a greater carcass hypertrophy conformation than females, the possible involvement of non-muscle-specific miRNAs in myogenesis that had not been yet thoroughly examined, and we report the pattern of expression of four non-muscle-specific miRNAs potentially involved in muscle hypertrophic pathway. In particular, we selected miR-27b because recently it has been recognized to have functional roles in cardiomyocytes [19] and skeletal muscle cells [20]. It has been reported that nuclear receptor peroxisome proliferator-activated receptor-γ (PPAR-γ) is targeted by miR-27b in the adult mouse heart and that mir-27b overexpression in primary cultured cardiomyocytes promoted hypertrophic cell growth through this way [21]. In muscle cells, miR-27b down-regulates Pax3 expression during myogenic differentiation [20] and MSTN in murine myoblast promoting proliferation [22]. Moreover, MEF2C, a muscle specific transcription factor, which plays essential roles during cardiac and skeletal myogenesis, has been demonstrated as another important target of miR-27b during heart development [19]. Although the specific functions and targets of miR-27 in hypertrophic pathway are largely unexplored, in this work we suggest that in Piedmontese cattle, MSTN is further regulated at post-transcriptional level by this miRNA. Mature miR-27 sequence, as predicted by Targetscan, is well conserved among a wide range of mammalian species, and our data are comparable to those obtained recently in mouse, where MSTN 3^′^UTR was demonstrated to be targeted by miR-27a/b [23]. Muscle samples were evaluated from male bovines based on S.E.U.R.O.P. grid classification. The S.E.U.R.O.P. system is currently in use for carcass classification in European Union. The carcass classification is done after slaughter, when the cleaned carcasses are halved and a trained classifier weighs and evaluates them with respect to fatness and fleshiness. We have shown the strong different expression of miR-27b when we compared animals from two opposite conformation such as “E” and “O”. On the contrary, the collected samples with carcass conformation E and U did not show a specific relationship with miR-27b (data not shown). We may argue that to observe differences in muscle hypertrophy we need to extend the analysis in a larger group of animals. The high ability of miR-27b to suppress MSTN 3^′^UTR lead us to hypothesize that different levels of expression of miR-27b may elucidate differences in muscle conformation. A 79% of 3^′^UTR MSTN knock down of luciferase activity is an exceptional down regulation compared to the 48% that Huang and colleagues have shown for miR-27a on murine MSTN 3^′^UTR. We suppose that in Piedmonte region the discrepancies among “E” and “U” subject are so small that these conformation differences are difficult to perceive at molecular level with a good statistical significance. Further studies are currently carrying out to elucidate this question.

## Conclusions

In summary, the important novel findings of our study are that exist a relationship between miR-27b and hypertrophic phenotype in bovine with a correlation between abundance of MSTN transcript/protein and miR-27b expression, and that MSTN gene is a target of miR-27b. Thus, our results provide the first evidence showing that MSTN-regulated miR-27b could play an important role in bovine skeletal muscle growth and hypertrophy. To our knowledge, this is the first study to report a link between double-muscled phenotype in Piedmontese bovine and miRNAs. These data indicate a specific role for miR-27b in the regulation of MSTN, which might be of great interest as selection marker to improve muscular hypertrophy.

## Methods

### Tissue sampling and processing

Immediately after slaughtering, muscle phenotype of animals was classified on visual estimation of muscle profiles through the use of S.E.U.R.O.P. assessment grid (S, super; E, excellent; U, abundant; R, good; O, fair; P, poor) (Review of the EU carcass classification system for beef and sheep (EPES 0708/01) Industry Consulting November 2008; available at http://archive.defra.gov.uk/evidence/economics/foodfarm/reports/carcaseclassification/FullVersion.pdf).

Samples of longissimus thoracis [24] were collected from 20 Piedmontese and 20 Fresian breed male bovine aged 16–23 months. Samples collection was performed with the authorization and under the supervision of representatives of the Veterinary Services of the Italian National Health Service branch of the Ministry of Health. Samples were placed in 10 volumes of RNAlater Solution (Ambion, Life technologies, Carlsbad, CA, USA) and transported to the laboratory in 1 hour at room temperature, then stored at 4°C overnight. After removal of the supernatant, samples were kept at −80°C for long-term storage.

### RNA extraction

Fifty milligrams of each sample were disrupted using a TissueLyser II (Qiagen) with stainless steel beads in 1 ml of TRI Reagent (Sigma-Aldrich, St. Louis, MO, USA); total RNA was purified from any residual genomic DNA with a DNAse I Recombinant RNAse free kit (Roche, Mannheim, Germany). The RNA concentration was determined by spectrophotometry (BioPhotometer, Eppendorf, Germany). The ratio of the optical densities measured at 260 and 280 nm were >1.9 for all RNA samples. cDNA was synthesized from 1 μg of total RNA using RT high Capacity cDNA Reverse Transcription kit (Applied Biosystems, Foster City, CA, USA) according to the manufacturer’s protocol. The cDNA was subsequently diluted in nuclease-free water and stored at −20°C.

### Quantitative expression of gene and miRNAs analysis

Sufficient cDNA (5 μg) was prepared in a single run to perform the q-PCR experiments for all the selected genes. To determine the relative amount of specific IGF1, IGF1R, PPP3CA, NFATc1, MEF2C, GSK3b, TEAD1 and MSTN transcripts, real-time quantitative PCR (qPCR) was performed using the MiniOpticonMJ detection system (Bio-Rad, Hercules, CA, USA). Primers for target and reference genes were designed on Bos taurus GenBank mRNA sequences using Primer 3 Software (version 4.0). Oligonucleotides were designed to cross the exon/exon boundaries to minimize the amplification of contaminant genomic DNA and were analyzed with the IDT tool (available at http://www.idtdna.com/scitools/scitools.aspx) for hairpin structure and dimers formation. Primer specificity was verified with BLAST analysis against the genomic NCBI database. Table 2 summarizes primer information including sequences, gene accession number and amplicon sizes. To establish primers efficiencies we have used the dilution method. MJOpticonMonitor3-Analysis software (version 3.1, Bio-Rad) calculates primers efficiency using the linear regression slope of the dilution series. Each primers set efficiency was comprised between 95% and 100%. Hypoxanthine phosphoribosyl-transferase 1(HPRT-1) gene was used as a reference gene for RNA concentration and reverse transcription efficiency [25].

**Table 2:** Primers for real-time quantitative PCR

**Gene**	**Accession number**	**5**^′^**-3**^′^**sequence**	**Amplicon size**
IGF1	NM_001077828	Fw: AGTTGGTGGATGCTCTCCAGT	115
		Rw: CACTCATCCACGATTCCTGTC	
IGF1R	NM_001244612	Fw: GGACGCAGTACGCCGTTTAC	187
		Rw: AGGGAGGGCGGGTTCCACTT	
PPP3CA	NM_174787	Fw: CAAGGCAATTGATCCCAAGT	204
		Rw: AGAATTGAAGCCCCCTCTGT	
NFATc1	NM_001166615	Fw: TACGAGCTGAGGATCGAGGT	139
		Rw: GAGGCTCGCTCTCCACATAG	
MEF2C	NM_001046113	Fw: CAGTCATTGGCTACCCCAGT	151
		Rw: GCGGTGTTAAAGCCAGAGAG	
GSK3b	NM_001101310	Fw: TTCCTTTGGAATCTGCCATC	233
		Rw: ACAGCTCAGCCAACACACAG	
TEAD1	XM_002693050	Fw: CTCAGGACAGGGAAGACGAG	134
		Rw: GGCTGCCCTGTTTGTATCAT	
MSTN	NM_001001525	Fw: GTGTTGCAGAACTGGCTCAA	256
		Rw: CAGCATCGAGATTCTGTGGA	

Primer sequences used for real-time quantitative PCR were designed on Bos taurus GenBank mRNA sequences using Primer 3 Software (version 4.0). Table summarizes primer information including sequences, gene accession number and amplicon sizes.

To quantify expression of mature miR-27b, miR-132, miR-186 and miR-199b-5p, 100 ng of total RNA were reverse transcribed using Taqman™ miRNAs reverse transcription kit, and subjected to real-time PCR using TaqMan™ miRNAs Assay kit (Applied Biosystems) according to the manufacturer’s protocol. MiR-16 was used to normalize the results [18]. qPCR parameters were as follows: cycle 1, 95°C for 3 minutes; cycle 2, 95°C for 15 seconds, 60°C 30 seconds for 40 cycles. Each reaction was run in triplicate, and a no-template control was included using water instead of cDNA. Differential expression was evaluated gene by gene by comparing the normalized Ct values (ΔCt) for all biological replicates between the two groups of samples. The fold change of expression of the transcript/miRNA was calculated (2^-ΔΔCT^). Data were expressed as fold-change compared to control samples [26].

### Western blot

Fifty milligrams of 20 samples of both bovine breeds were washed with ice-cold PBS and were disrupted in lysis buffer (20 mM Tris, pH 7.4, 150 mM NaCl, 5 mM EDTA, 1% Triton X-100) with 1 mM phenylmethylsulfonyl fluoride, 10 mM NaF, 1 mM Na_3_VO_4_, and protease inhibitor cocktail (Sigma-Aldrich) using a Dounce glass homogenizer. The homogenization was operated manually and tissue was disrupted by forcing it between the pestle and tube wall. Protein lysates were cleared of cellular debris by centrifugation at 4°C for 10 minutes at 12000 g. Total proteins were quantified using Bio-Rad protein assay (Bio-rad) with bovine serum albumin (BSA) as standard. Twenty micrograms of total protein were resolved in 10% SDS-PAGE gels, and transferred to Hybond ECL Nitrocellulose Membranes (Amersham Biosciences, Germany). Western blots were performed with polyclonal anti-MSTN and monoclonal anti-α-tubulin (Sigma-Aldrich) antibodies (1:1000). The anti-MSTN antibody has been generated from the 191–205 myostatin peptide, that is homologous to the bos taurus one and is far from the Piedmontese myostatin mutation site. Using this antibody we have detected a single protein band (at about 29 kDa mature MSTN). No other band referable to precursor MSTN forms were visualized. A protein extract of Huvec cell line was used as positive control according to Sigma–Aldrich staining (data not shown). Proteins were visualized with horseradish peroxidase–conjugated secondary antibodies (1:2000) and ECL Plus Western Blotting Detection System (Euroclone, Pero, Italy). Blots were exposed to CL-X Posure Film Clear Blue X-Ray Film (Pierce, Rockford, IL, USA). Densitometric quantification of MSTN intensity of Western blot bands was carried out by using ImageJ software (National Institutes of Health, Bethesda, MD). Developed films were scanned as JPEG in 8 bit gray-scale format at 600 dpi and the pixel intensities within each band of MSTN and α-tubulin were measured as area under curve (AUC). For each muscle sample, to quantify the MSTN bands obtained, the AUC of MSTN was corrected for the AUC of corresponding α-tubulin. MSTN abundance was calculated as a ratio between AUC of MSTN band and AUC of the housekeeping protein band (MSTN/α-tubulin) in order to correct for small α-tubulin abundance variations in lanes.

### DNA constructs

The 3^′^-UTR of the MSTN transcript was amplified from genomic Piedmontese bovine DNA by PCR using forward 5^′^- GGTCTATATTTGGTTCATAGCTTCC -3^′^and reverse 5^′^- TCTTTCAAAAAAGGTGAAAACAC -3^′^ primers, cloned into the Cloning vector pJET (Clone Jet PCR Cloning kit, Fermentas), and sequence was confirmed. The predicted bovine miR-27b seed-site was mutated with mutagenic primers by using the Quick Change Multisite mutagenesis kit (Stratagene, Waldbronn, Germany); mutations were confirmed by sequence analysis. The wild-type and mutated 3^′^-UTRs were subcloned into the NotI–XhoI site of the psicheck-2 vector (Promega, Madison, WI, USA) (see Additional file 1).

### Transfections

293T cell line was purchased from the American Type Culture Collection (ATCC, Rockville, MD, USA). The cell line was maintained in Dulbecco modified Eagle medium (DMEM) (Sigma-Aldrich) supplemented with 10% fetal bovine serum (FBS) (Euroclone), and antibiotics (100 U/mL penicillin and 100 lg/L streptomycin) (Euroclone) at 37°C in a 5% CO_2_, humidified atmosphere. 293T were transfected at 60% confluence. Cells were seeded at 1x10^4^ cells per well of a 24-well plate and grown for 24 h. A total of 300 ng of the appropriate psicheck-2 luciferase reporter construct was cotransfected with 15 nM final concentration of either double-stranded RNA oligonucleotides designed to mimic miR-27b and miR-142 molecules (Ambion) using Metafectene (Biontex Laboratories, Planegg, Germany) according to the manufacturer’s protocol. Twenty-four hours after transfection, the medium was changed and cells were grown for an additional 24 h before assay.

### Luciferase assay

Firefly and Renilla luminescent signals arising from psicheck-2 transfected cells were quantified according to the manufacturer’s instructions using Dual Luciferase assay system (Promega) with a VICTOR Multilabel Counter luminometer (PerkinElmer, Waltham, MA). All values are given relative to transfections with the appropriate negative control.

### Statistical analysis

Statistical significance was assessed by Mann–Whitney *U*-test given the violation of Student’s t- test required assumption of normality as assessed through Kolmogorov-Smirnov test; considering the limited sample size which impairs *t*-test robustness, the non parametric alternative was chosen for the final between group comparison. For the same reason the non parametric test of Spearman correlation was applied to miR-27b expression and MSTN transcript/protein abundance in Piedmontese breed. Data were expressed as mean ± SD. Differences were considered as significant at a level of p < 0.05. All statistical analysis mentioned above were performed using GraphPad software InStat.

## Competing interests

The authors declare that they have no competing interests.

## Authors’ contributions

SM carried out the biological experiments, analyzed the data, and drafted the manuscript. EM and PA participated in study design and provided samples. MB conceived the study and helped to draft the manuscript. All authors read and approved the final manuscript.

## Supplementary Material

Additional file 1: **Psi-check2 reporter vector containing native or mutant MSTN 3**^′^**-UTR.** Vector contains Renilla luciferase reporter gene, hRluc, which is used to monitor changes in expression of 3^′^-UTR as the result of miRNA binding. The firefly luciferase reporter gene, hluc, is used to correct luciferase signal for transfection efficiency. The 1500-bp MSTN 3^′^-UTR sequence containing miR-27b consensus sequence (wilde type or mutated) was inserted into the vector at the 3^′^ end of the hRluc reporter gene. (TIFF 28 kb) Click here for file
