# Supplementary figures and images for: Functional effect of mir-27b on myostatin expression: a relationship in piedmontese cattle with double-muscled phenotype
Source: BMC Genomics. 2013 Mar 19;14:194. doi: 10.1186/1471-2164-14-194 (PMC3605361; doi:10.1186/1471-2164-14-194)

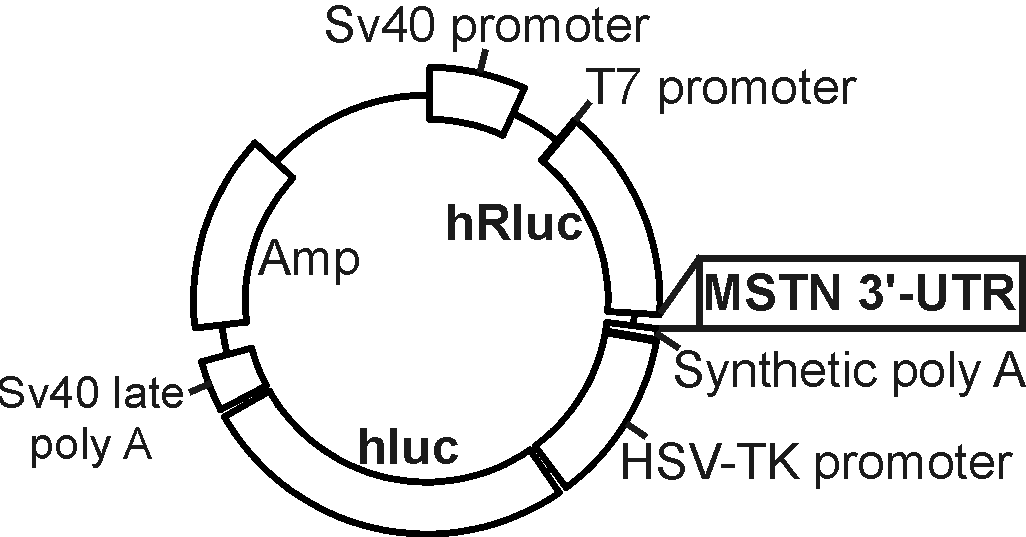

Supplement: Additional file 1: — Psi-check2 reporter vector containing native or mutant MSTN 3′-UTR. Vector contains Renilla luciferase reporter gene, hRluc, which is used to monitor changes in expression of 3′-UTR as the result of miRNA binding. The firefly luciferase reporter gene, hluc, is used to correct luciferase signal for transfection efficiency. The 1500-bp MSTN 3′-UTR sequence containing miR-27b consensus sequence (wilde type or mutated) was inserted into the vector at the 3′ end of the hRluc reporter gene. (TIFF 28 kb) [file 1471-2164-14-194-S1.tiff]
